# *Cicinnus chambersi*: a new species of sack-bearer moth (Lepidoptera, Mimallonidae, Cicinninae) from southeastern Arizona, USA

**DOI:** 10.3897/zookeys.931.50203

**Published:** 2020-04-30

**Authors:** Ryan A. St Laurent, Lawrence E. Reeves, Akito Y. Kawahara

**Affiliations:** 1 Department of Biology, University of Florida, Gainesville, FL 32611, USA University of Florida Gainesville United States of America; 2 McGuire Center for Lepidoptera and Biodiversity, Florida Museum of Natural History, University of Florida, 3215 Hull Road, Gainesville, FL 32611–2710, USA University of Florida Vero Beach United States of America; 3 Entomology and Nematology Department, University of Florida, 1881 Natural Area Dr., Gainesville, FL 32608, USA University of Florida Gainesville United States of America; 4 Florida Medical Entomology Laboratory, Institute for Food and Agricultural Sciences, University of Florida, 200 9th St. SE, Vero Beach, Florida 32962, USA University of Florida Vero Beach United States of America

**Keywords:** *Cicinnus chambersi* sp. nov., Mimallonoidea, Sky Islands, taxonomy

## Abstract

A new species of cicinnine Mimallonidae, *Cicinnus chambersi***sp. nov.**, is described from the Sky Islands Region of southern Arizona, USA. The new species is closely related to *C. mexicana* (Druce), type locality Veracruz, Mexico, based on morphology and genetics. The other *Cicinnus* species known from the United States, the common *C. melsheimeri* (type locality Pennsylvania, USA) is morphologically and genetically distinct from both *C. chambersi* and *C. mexicana*. The new species is compared to *C. mexicana* and *C. melsheimeri*, as well as other Mexican *Cicinnus*. The life history of *C. chambersi* is unknown, but its description should facilitate future studies on this rarely reported North American mimallonid, a species which may have only recently become established in the United States. *Cicinnus chambersi* is the fifth known Mimallonidae species from the United States, and the first described from the country in nearly half a century.

## Introduction

Mimallonidae are a family of approximately 300 species of moths endemic to the New World, with the vast majority of species found in Central and South America (St Laurent and Kawahara 2019). The most recent revision of North American Mimallonidae by Franclemont (1973) recognized four species from the United States: *Lacosoma arizonicum* Dyar, 1898, *L. chiridota* Grote, 1864, *L. elassa* (Franclemont, 1973), and *Cicinnus melsheimeri* (Harris, 1841). Franclemont (1973) treated three genera in North America: *Lacosoma* Grote, 1864, *Naniteta* Franclemont, 1973, and *Cicinnus* Blanchard, 1852. Apart from the synonymization of *Naniteta* with *Lacosoma* by St Laurent et al. (2018), no major taxonomic work has since been carried out focusing on the Mimallonidae of the United States. Recent phylogenetic and systematic research has ultimately led to a major reassessment of the intrafamilial classification of Mimallonidae, resulting in the recognition of various subfamilies and tribes, new genera, and a multitude of new combinations (St Laurent et al. 2018, 2020; St Laurent and Kawahara 2019). This systematic body of work has therefore provided an understanding of the relative relationships of the two valid genera of Mimallonidae found in the United States and Canada, with *Lacosoma* being the type genus of Lacosominae and *Cicinnus* the type genus of Cicinninae; which are relatively distantly related within Mimallonidae.

Two mimallonid species inhabit the eastern United States and extreme southern Ontario east of the Great Plains: *L. chiridota* and *C. melsheimeri*. Although the majority of *C. melsheimeri* records are from the eastern United States, this species is quite widespread in the Rocky Mountains, although it is rarely collected in the region. A single, predominantly Mexican species reaches its northernmost extent in southern Arizona: *L. arizonicum* (Franclemont 1973; Powell and Opler 2009). *Lacosoma elassa* is only known from two specimens collected in Brownsville, Texas (Franclemont 1973).

In southern Arizona, *L. arizonicum* is a somewhat regularly collected, late night-flying denizen of mid-elevation oak forests, and its life history was recently published (Powell and Opler 2009; St Laurent et al. 2017). *Cicinnus* in contrast, is poorly represented in collections from Arizona and from the western United States in general. There is sparse literature and few collection records of *C. melsheimeri* from the Rocky Mountains and northern Arizona, New Mexico, western Texas and Mexico. Reports from southern Arizona are limited to recent online records (e.g., https://www.BugGuide.net). We discuss *Cicinnus* in southern Arizona, recognize the presence of a species there that is more closely related to the widespread Mexican species *C. mexicana* (Druce, 1898) than to *C. melsheimeri*, and describe it as new. This is the first new species of Mimallonidae described from North America since the 1970s, and only the second belonging to *Cicinnus* found in the United States.

The Sonoran Desert’s Sky Islands Region is located at a biogeographic crossroads at the convergence of several biotic zones. Patterns of biodiversity are influenced by the ecological communities of the Sonoran and Chihuahuan Deserts, the Rocky Mountains, the Great Plains, the Sierra Madre and the Neotropics (Baynham 2012). The Sky Islands Region is characterized by an archipelago-like series of about 65 mountain ranges, each harboring isolated mid- and high-elevation oak and pine forests, surrounded by a sea of arid and semi-arid desert and grassland (Moore 2015). The onset of the summer monsoon influences the activity patterns of much of the region’s biodiversity, prompting growth and reproduction in many plants, and prompting adult emergence and activity among many insect species (Ingram and Brusca 2015).

The combination of location, at the interface of biotic zones, the complex topography of the Sky Islands, and relative isolation of mountain range islands promotes unique ecological communities with high species diversity and endemism (Van Devender et al. 2013). Although the arthropod diversity of the region is relatively understudied (Moore et al. 2013), the moths of southeastern Arizona have been collected and documented for decades, and the region contains several classic collecting sites for moths and other insects including Box Canyon, Harshaw Creek, Madera Canyon, Peña Blanca Lake, and Ruby Road, among others. Many of these sites (e.g., Harshaw Creek, Madera Canyon) have been heavily collected by both amateur and professional entomologists for decades, with novel taxa being described from these localities (e.g., Lemaire et al. 1992; Donahue 1993).

## Materials and methods

### Taxonomic methods

All dissections performed for this study followed Lafontaine (2004) in methodology, with genitalia stored in glycerol-filled microcentrifuge vials. Genitalia of *Cicinnus* are incredibly intricate, complex, three dimensional structures, and therefore slide-mounting was not conducted in order to preserve the natural structural integrity. Labels of the holotype are given verbatim, with forward slashes used to denote separate labels.

Specimens examined are deposited in the collections listed below. Figures in this paper were created with Adobe Photoshop as part of the Creative Cloud (Adobe 2019), and maps were built using SimpleMappr (Shorthouse 2010). The following collections were used for specimens pertinent to the present study:

**AMNH**American Museum of Natural History, New York, New York, USA;

**BME** Bohart Museum of Entomology, University of California, Davis, California, USA;

**BWC** B. Walsh Private Collection, Tucson, Arizona, USA;

**CJM** Collection of José Monzón, Guatemala;

**CRAS** Research collection of R. St Laurent, Gainesville, Florida, USA;

**CUIC**Cornell University Insect Collection, Ithaca, New York, USA;

**MCZ** Museum of Comparative Zoology, Harvard University, Cambridge, Massachusetts, USA;

**MGCL** McGuire Center for Lepidoptera & Biodiversity, Gainesville, Florida, USA;

**PJD** Collection of Paul J. Dennehy, Pennsylvania, USA;

**VOB** Becker Collection, Camacã, Bahia, Brazil;

### Molecular phylogenetics

We refer to the anchored hybrid enrichment (AHE) Mimallonidae phylogeny of St Laurent et al. (2020). While we do not conduct AHE analyses here, we discuss relationships of *Cicinnus* in this aforementioned work in order to bolster our understanding of the phylogenetic relationships of North American *Cicinnus*. In the present study we utilize sequences of the mitochondrial “barcoding” gene, cytochrome c oxidase subunit I (COI) in combination with morphology (Hebert et al. 2003). We sequenced 11 samples of Cicinnus*de novo*, and downloaded additional publicly available Cicinnini samples from BOLD (Barcode of Life Datasystems) (Ratnasingham and Hebert 2007) to compliment the material that we sequenced for this study. For *de novo* sequence data generation, one or two legs were removed from recently collected museum specimens and submitted to the Smithsonian Institution, Washington D.C. as part of the Smithsonian Institution DNA Barcode Network. Sequence assembly was conducted in Geneious v. 2019.2.1, and alignment performed with MUSCLE in AliView (Larsson 2014).

**Figure 1.**
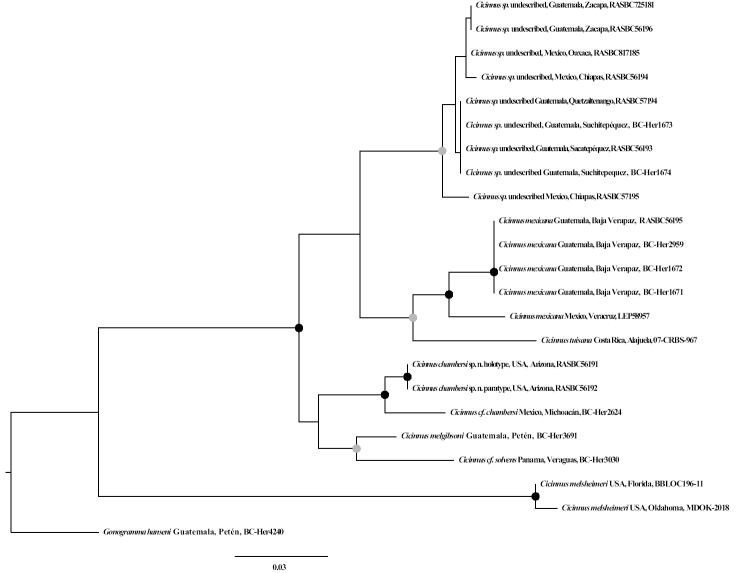
Maximum likelihood phylogenetic tree inferred with IQ-TREE based on the COI marker, rooted to *Gonogramma hanseni*. Black circles indicate SH-aLRT/UFBoot of 80/95 or greater for both values, and gray circles indicate SH-aLRT/UFBoot of 80–95 for both values. The scale bar represents expected number of nucleotide substitutions per site. See Suppl. material 1: Figure S1 for complete support values.

Maximum Likelihood (ML) phylogenetic analyses of unpartitioned COI data was performed using IQ-TREE v. 1.6.10, with branch supports reported as 1,000 Ultrafast Bootstraps (UFBoot) and SH-aLRT as a secondary measure of support (Nguyen et al. 2015; Hoang et al. 2018). The most optimal model of nucleotide evolution was selected by ModelFinder within IQ-TREE (Kalyaanamoorthy et al. 2017), and the TIM2+F+G4 model was used in 1,000 independent tree searches in IQ-TREE, the best scoring tree was used in Fig. 1 (and Suppl. material 1: Figure 1) and all discussions. Each of the 1,000 independent IQ-TREE runs also utilized UFBoot and SH-aLRT supports, as well as the -bnni option to further optimize UFBoot trees using Nearest Neighbor Interchange (NNI) in order to alleviate inherent biases of the UFBoot.

All sequence data provided by this study will be made available on GenBank, with applicable accession numbers provided in Suppl. material 2: Table S1. A COI FASTA alignment and tree file, including all taxa utilized in this study, are provided as Suppl. material 4: File S2 and Suppl. material 5: File S3 respectively.

## Results and discussion

### Cicinnus chambersi sp. nov.

Taxon classificationAnimaliaLepidopteraMimallonidae

DC69B8CA-CF5F-550D-A452-3BAD248C8C5B

http://zoobank.org/39AED117-04A1-434F-B3B8-5AD826A3D52E

Figures 2 , 3 , 6 , 7 , 10 , 11 , 15 , 16 , 21

#### Type material.

***Holotype*. United States of America** – **Arizona** • Arizona: Santa Cruz. Co., Peña Blanca Lake, Pajarito Mtns., Coronado NF; 750 W MV, 1000W MH, 31.402057, -111.084236, 21.VII.2015; leg. L.E. Reeves/ St Laurent dissection 2-20-17:1 *Cicinnus* sp./ St Laurent barcode 2-20-17:1 [barcode unsuccessful]/ St Laurent BC 5-6-19:1 [second barcode attempt]/ Holotype ♂ *Cicinnus chambersi* St Laurent, Reeves, Kawahara, 2020 [red label]/ (MGCL).

**Figures 2–5.**
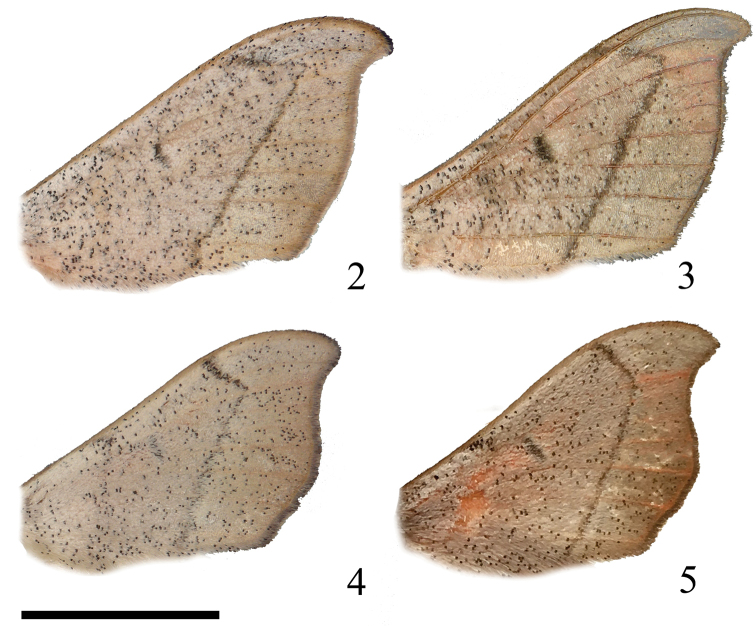
*Cicinnus* adult male forewing comparison showing the acuter angle of the postmedial line near the apex in *C. chambersi* than in *C. melsheimeri***2***C. chambersi* holotype, Arizona **3***C. chambersi* paratype, Arizona **4***C. melsheimeri*, Florida **5***C. melsheimeri*, New Jersey. Scale bar: 1 cm.

***Paratypes*.** (3 ♂, 2 ♀ total) **United States of America** – **Arizona** – **Cochise County** • 1 ♀; Copper Canyon, Huachuca Mts; 31.363, -110.300; 6,000 ft [1,828 m]; 4.VII.2018; C.W. Melton [leg.]; photo ID no. 18070692, St Laurent dissection: 5-9-19:1; (MGCL). – **Santa Cruz County** • 1 ♂, 1 ♀; California Gulch, Pajarito Mountains; 31.422N, 111.245W; 3800 ft [1,158 m]; 27.VII.2017; J.B. Walsh leg.; MV/UV; (BWC) • 1 ♂; Peña Blanca Lake/ Ruby Rd area; 31°23'16"-24'N, 111°05'25"-07'W; 2–4.VIII.2017; James Adams & Lance Durden; light traps, LEP-58833 [MGCLAHE voucher number and St Laurent dissection number], St Laurent BC 5-6-19:2 [barcode]; (MGCL) • 1 ♂; Peña Blanca Canyon; 31.3844N, 111.0935W; 3.VIII.2017; P. Dennehy leg.; (PJD). Paratypes with the following yellow label: Paratype ♂/ ♀ *Cicinnus chambersi* St Laurent & Reeves, 2020.

**Figures 6–9.**
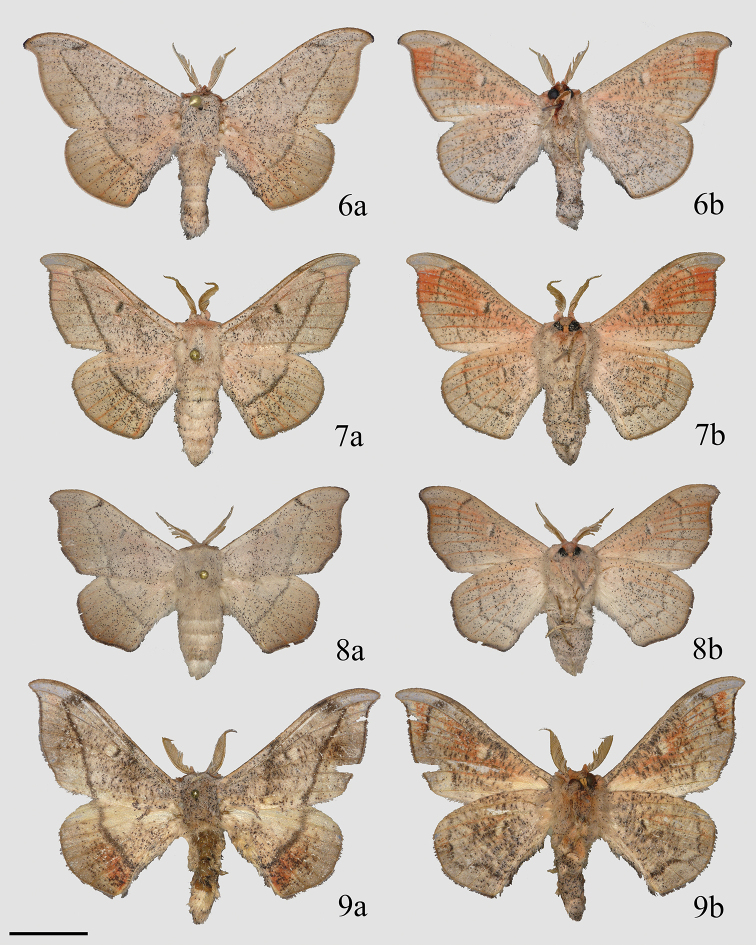
Adult ♂ *Cicinnus***a** dorsal **b** ventral **6***C. chambersi* holotype, USA, Arizona, Santa Cruz Co., Peña Blanca Lake, Pajarito Mtns., Coronado National Forest (MGCL) **7***C. chambersi* paratype, USA, Arizona, Santa Cruz Co., Peña Blanca Lake/ Ruby Rd area (MGCL) **8***C. melsheimeri*, USA, Florida, Alachua Co., Micanopy (MGCL) **9***C. chabaudi*, Mexico, Oaxaca, ca. 15 km SE San Martín Huamalulpan, Cabañas Yucunuvichi (MGCL). Scale bar: 1 cm.

#### Additional specimens

[**not included in type series]. United States of America** – **Arizona** – **Santa Cruz County** • 2 ♂, 1 ♀; California Gulch; 31°25'18.33"N, 111°14'40.02"W; 3,790 ft [1,155 m]; 23.VII.2015 [2 ♂], 21.VII.2017 [1 ♀]; E. Rand leg. (Coll. E. Rand, Arizona) • 1 ♂; Peña Blanca Canyon; 31°23'18.38"N, 111°5'33.00"W; 3895 ft [1,187 m]; 17.VII.2009; E. Rand leg. (Coll. E. Rand, Arizona) • 1 ♂; Jct. FR 49 & FR 812; 31°27'54.88"N, 110°43'9.94"W; 4960’ [1,512 m]; 8.VII.2010; E. Rand leg. (Coll. E. Rand, Arizona).

#### Photographed individual

**Figure 10.**
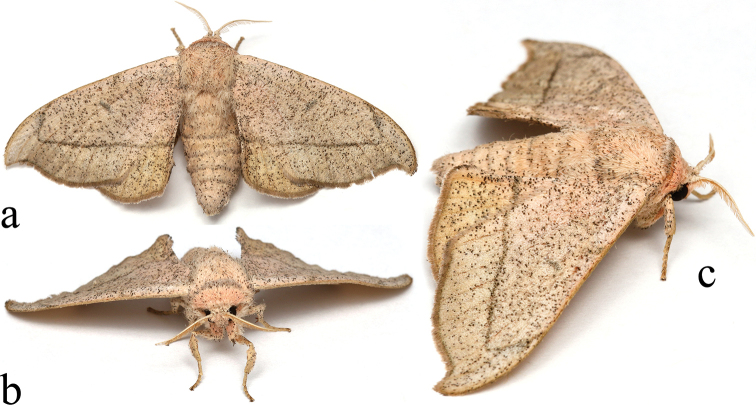
Living adult ♀ *Cicinnus chambersi*. USA, Arizona, Pima Co., Box Canyon, photographed by Salvador Vitanza, Entomologist (used with permission) **a** dorsal **b** anterior **c** lateral.

[**not collected and not included in type series]. United States of America** – **Arizona** – **Pima County** • 1 ♀; Box Canyon; 31.799198, -110.798744; photographed by Salvador Vitanza, Entomologist (Identifier) at APHIS-PPQ, Arizona (Fig. 10).

#### Diagnosis.

In southern Arizona, there are no other moths with which this species could be confused, the only other congener found in the United States, *C. melsheimeri*, has not been found to be sympatric with *C. chambersi*, but occurs farther north in mountainous northern Arizona, north of the Mogollon Rim, and northeast into central and northern New Mexico. Because *C. chambersi* and *C. melsheimeri* are both found in Arizona, we compare them here, although they are not each other’s closest relatives within *Cicinnus* (see remarks later).

*Cicinnus melsheimeri* is a somewhat variable species, usually with brown shaded regions along the wing margins, in comparison, *C. chambersi* is more consistently uniformly pink in coloration with a homogenous ground color. The apex of the forewing of *C. chambersi* is much sharper than in *C. melsheimeri*, and the postmedial line of the forewing more distinctly forms a right or acute angle near the apex of the forewing, whereas this same angle is more obtuse in *C. melsheimeri* (see Figs 2–5, where males are shown, the same distinction is observed in females as well. Line thickness and development of discal spots are rather variable characteristics in both species).

Genitalia of the two species of *Cicinnus* in Arizona are structurally very distinct, although they both have the characteristics deemed apomorphic of *Cicinnussensu stricto* as defined by St Laurent and Kawahara (2019). Fundamental differences in male genitalia of the two species in question are: *C. melsheimeri* bears elongated vincular arms (Fig. 17) which are naturally held along a ventral channel on the valvae, *C. chambersi* and all other *Cicinnus* lack vincular arms; the valvae of both *C. melsheimeri* and *C. chambersi* are mostly membranous, but in *C. melsheimeri* they are even more so and are dorso-ventrally wider than laterally, whereas in *C. chambersi* the valvae are squarer with a more substantially sclerotized costal half; the juxtal complex in *C. chambersi* is bifurcated on either side of the phallus but ends in a singular upward curling terminus on either side of the phallus in *C. melsheimeri*. Female genitalia of *C. chambersi* are typical of *Cicinnus*, differing from those of *C. melsheimeri* by the narrower dorsal projection of the VIII segment, more well-developed anterior and posterior apophyses, a ductus bursae that is at least five times longer in length, and an elongated corpus bursae that is roughly four times the length of that of *C. melsheimeri* (compare Figs 21, 23).

The differences between *C. chambersi* and the other Mexican *Cicinnus* species are less obvious. The only names currently applied to similar Mexican species are *C. chabaudi* Dyar, 1914 (Figs 9, 18), and *C. mexicana* (Figs 14, 20), the latter which includes at least two cryptic species (see remarks and additional discussion below). *Cicinnus chabaudi* is restricted to arid south-central Mexico in the vicinity of the Distrito Federal, this species is more darkly maculated than *C. chambersi* with deep reddish-brown anal areas of the hindwings. *Cicinnus mexicana* is a variable species, often with deep reddish-brown submarginal areas of the forewings and straighter wing margins (all previously discussed *Cicinnus* species have convex forewing margins), but some populations are lighter with more convex forewing margins. The male genitalia of these various Mexican *Cicinnus* are quite similar with only minor differences from species to species with the most useful characters in this particular group (valvae shape and juxtal complex) displaying intraspecific variation (compare *C. chambersi* to the others: Figs 15, 16 to 18–20; and the two *C. chambersi* specimens figured in Figs 15, 16 to each other). *Cicinnus chambersi* however, can be recognized by the squarer shape of the valvae, which are generally more rounded and splayed upward in other Mexican *Cicinnus*. The intricacies of the taxonomy of *Cicinnus* in Mexico is discussed below in the remarks and further discussion sections.

**Figures 11–14.**
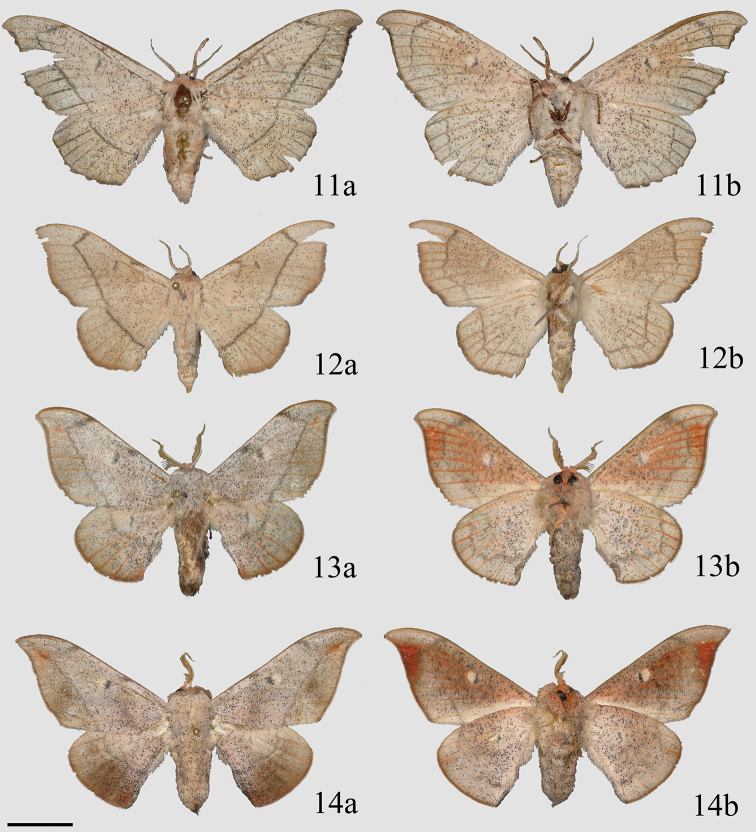
Adult *Cicinnus***a** dorsal **b** ventral **11***C. chambersi* ♀ paratype, USA, Arizona, Cochise Co., Copper Canyon, Huachuca Mts, 1828 m (MGCL) **12***C. melsheimeri* ♀, USA, Texas, Cameron Co., Brownsville (MGCL) **13**C. undescribed near mexicana ♂, Guatemala, Zacapa, Sierra de las Minas, N Rio Hondo, E San Lorenzo, Cerro Monos env., 2243 m (MGCL) **14***C. mexicana* ♂, Guatemala, Baja Verapaz, SE Purulhá, Ranchitos de Quetzal, Parque Ecológico Gucumatz, 1660 m (MGCL). Scale bar: 1 cm.

**Figures 15, 16.**
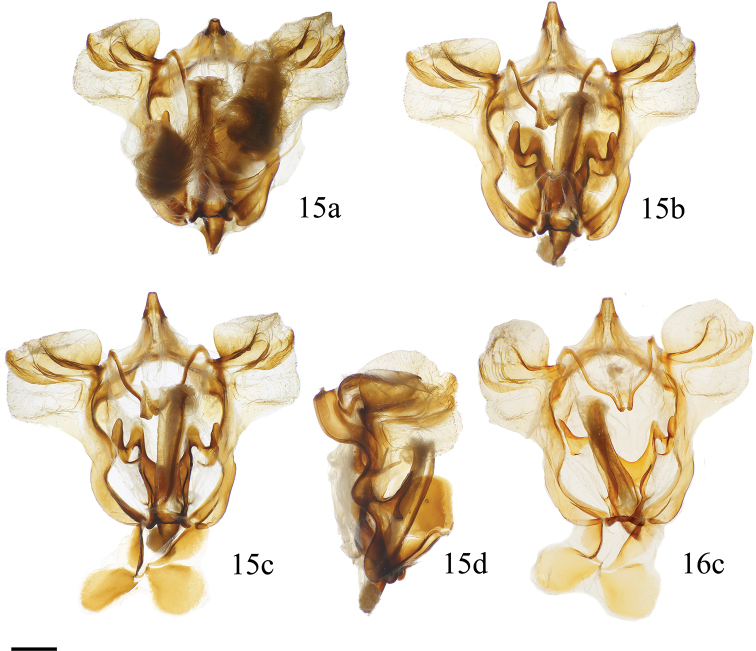
*Cicinnus chambersi* ♂ genitalia **a** ventral, vinculum extension in natural position, deciduous setae intact **b** ventral, vinculum extension in natural position, deciduous setae removed **c** ventral, vinculum extension held open **d** lateral **15** holotype, USA, Arizona, Santa Cruz Co., Peña Blanca Lake, Pajarito Mtns., Coronado National Forest, St Laurent dissection: 2-20-17:1 (MGCL) **16** paratype, USA, Arizona, Santa Cruz Co., Peña Blanca Lake/ Ruby Rd area, St Laurent dissection: LEP58833 (MGCL). Scale bar: 1 mm.

**Figures 17–20.**
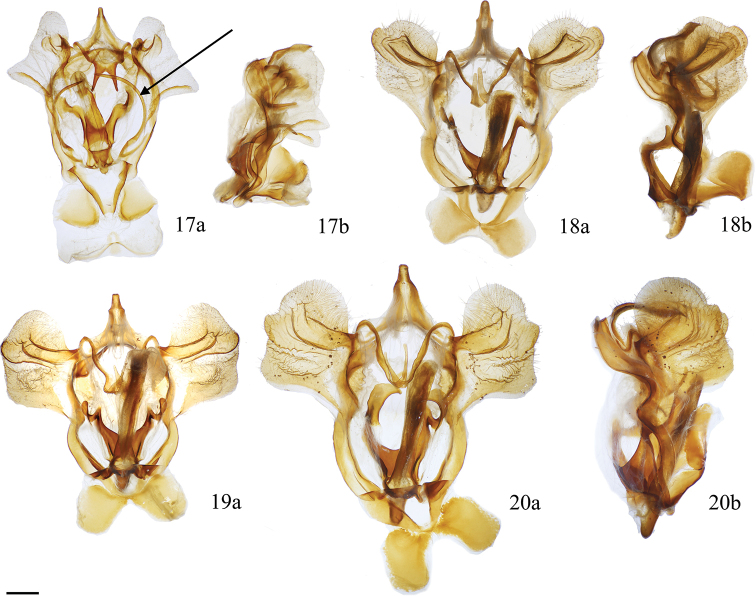
*Cicinnus* ♂ genitalia **a** ventral, vinculum extension held open **b** lateral **17***C. melsheimeri*, USA, Ohio, Geauga Co., Thompson Township, St Laurent dissection: 8-10-18:1 [arrow denotes vincular arms which are unique to *C. melsheimeri* among *Cicinnus*] (MGCL) **18***C. chabaudi*, Mexico, Oaxaca, ca. 15 km SE San Martín Huamalulpan, Cabañas Yucunuvichi, St Laurent dissection: 8-10-18:3 (MGCL) **19**C. undescribed near mexicana Guatemala, Zacapa, Sierra de las Minas, N Rio Hondo, E San Lorenzo, Cerro Monos env., 2243 m, St Laurent dissection: 7-25-18:1 (MGCL) **20***C. mexicana*, Guatemala, Baja Verapaz, SE Purulhá, Ranchitos de Quetzal, Parque Ecológico Gucumatz, 1660 m, St Laurent dissection: 7-25-18:4 (MGCL). Scale bar: 1 mm.

#### Description.

**Figures 21–23.**
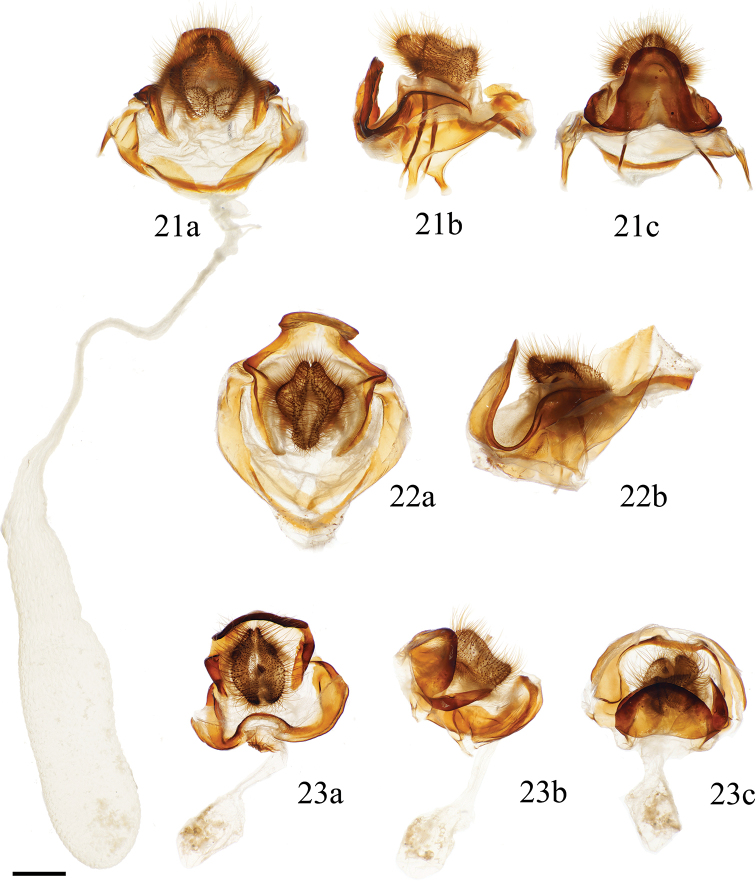
*Cicinnus* ♀ genitalia **a** ventral **b** lateral **c** dorsal **21***C. chambersi* paratype, USA, Arizona, Cochise Co., Copper Canyon, Huachuca Mts, 1828 m, St Laurent dissection: 5-9-19:1 (MGCL) **22**C. undescribed near mexicana, Mexico, Chiapas, San Cristobal de las Casas env., nr. Hotel Flores, 2415 m, St Laurent dissection: 5-6-19:4 [note: ductus and corpus bursae not shown, but highly elongate as in Fig. 22] (MGCL) **23***C. melsheimeri*, USA, Indiana, Brown Co., Brown County State Park, St Laurent dissection: 5-11-19:1 (MGCL). Scale bar: 1 mm.

**Male. *Head***: Coloration pinkish beige with an ample speckling of dark brown petiolate scales. Antennae pale yellow with a covering of beige scales, occasionally speckled with darker brown scales, bipectinate to tip, distal quarter of pectinations dramatically shorter than basal three quarters of pectinations. Eyes very large, comprising more than two thirds area of head. Labial palpus exceedingly short, not extending beyond frons, coloration as for head though with darker gray scales dorsally; labial palpus apparently three-segmented though distalmost segment miniscule. ***Thorax***: Dorsally light beige with profuse speckling of dark brown petiolate scales, prothorax lighter in color, pinker, ventrally thorax as above. ***Legs***: Coloration mostly as for thorax. Tibial spurs small, about as long as one quarter length of first tarsomere. ***Forewing dorsum***: Forewing length: 20–22 mm, avg.: 21 mm; wingspan: 39–45 mm, *N* = 3. Triangular, apex sharply falcate, outer margin mostly convex except for concavity below apex and slight tornal concavity. Ground color same light beige as thorax, with underlying pink hue throughout, profusely speckled with dark brown petiolate scales which are less densely distributed submarginally. Antemedial line very faint, usually nonexistent but if present diffuse and wavy. Postmedial line fine, almost always nearly straight, well-defined, dark brown, perpendicularly angled toward costa after passing Rs4, line thickness variable but comparatively thicker near costa. Entire wing nearly concolorous except for postmedial line and discal spot though coloration grayer rather than pink along costa, especially apically. Discal spot variably developed ranging from faint comma-like mark to well-developed gray-brown oval situated at distal margin of discal cell. Fringe darker brown than ground color of wing. ***Forewing ventrum***: Ground color similar to dorsum but suffused with bright orange-red especially medially and along veins, basally wing much pinker than dorsally. Bright orange-red patch of scales present submarginally between Rs3–M3. Postmedial line weakly defined, consisting of dentate, convex line that is neither straight nor distinctly angled toward costa. Discal spot may be more well-defined than on dorsum. ***Hindwing dorsum***: Rounded, coloration and patterning as for forewing dorsum, but antemedial line absent, postmedial line outwardly convex, discal spot less defined. ***Hindwing ventrum***: Follows similar pattern as forewing ventrum, postmedial line convex and more interrupted by veins than on dorsum, discal spot weakly developed. ***Abdomen***: Robust, extending beyond anal angle of hindwing, coloration mostly as for thorax. Sternite of VIII anteriorly and posteriorly concave, with pair of short protuberances, one on either side of posterior concavity. ***Genitalia***: (Figs 15, 16) *N* = 2. Complex, though typical of *Cicinnus*. Vinculum rectangular with pair of ventral apodemes. Tegumen triangular but not particularly distinct from uncus. Uncus simple, triangular, ventrally with blunt apex, but appearing sharp laterally due to distal flattening. Gnathos originating from between base of uncus and dorsal junction of valvae with vinculum, gnathos swoops downward from origination point to central location below uncus, distally gnathos extends as pair of fingerlike projections. Valvae mostly membranous with sclerotization restricted to upper region of valvae, particularly along mesal bar that extends outward along length of valvae, valvae rectangular in shape, small relative to remainder of genitalia. Juxta fused to phallus, extending laterally on either side of phallus with pair of sclerotized projections curling upward. Phallus cylindrical, mostly membranous. Vesica bag-like. Base of vinculum extends outward as two heavily sclerotized arms ending in bilobed, thin, sclerotized structure which is naturally curled upward covering genitalia, within which densely packed setae stored (Fig. 15a). **Female. *Head***: As for male in coloration but antennae appearing longer, comparatively thinner due to much shorter pectinations along length, pectinations gradually decrease in length from base to tip of antenna. ***Thorax***: As for male. ***Legs***: As for male but tibial spines much longer, at least double length of those of male. ***Forewing dorsum***: Forewing length: 28 mm; wingspan: 60 mm, *N* = 1. As for male but wing shape slightly wider and more elongated, hue of wing lighter than in male. ***Forewing ventrum***: Similar to dorsum, lacking any deep orange-red coloration of male. Antemedial line absent, postmedial line weakly defined, consisting of dentate, convex line. Discal spot about as well-defined as on dorsum. ***Hindwing dorsum***: As for male, coloration barely lighter. ***Hindwing ventrum***: Follows similar pattern as forewing ventrum, postmedial line convex, discal spot nearly absent. ***Abdomen***: As for male, but more robust, coloration mostly as for thorax. ***Genitalia***: (Fig. 21) *N* =1. Tergite VIII forms smooth, heavily sclerotized, posteriorly directed tongue-like extension which nearly reaches distalmost apex of papillae anales. Apophyses anteriores roughly one third length of apophyses posteriores, much thicker, stouter. Lamella antevaginalis weakly sclerotized and split mesally, lamella postvaginalis a more heavily sclerotized band but not as wide as lamella postvaginalis. Ductus bursae very long, narrow, about twice length of remainder of genitalia. Corpus bursae narrow, tubular, longer than ductus bursae; ductus and corpus bursae together about as long as entire abdomen. Papillae anales widest mesally, distally pinched together and somewhat projected upward, overall densely covered in elongate setae.

#### Biology.

The life history of *C. chambersi* is unknown, but we expect the larvae feed on oaks (*Quercus* spp. Linnaeus) as do all Mimallonidae in Canada and the United States for which larval hosts are known. *Cicinnus chambersi* appears to be a denizen of mid-elevation oak-dominated habitats of the Sky Island Region. The type series and other examined specimens were collected at elevations ranging from 1,155 m to 1,828 m, all within the oak belt of sky island mountain ranges (Baynham 2012). This narrow elevation range suggests possible specialization on certain oak species that are also restricted in elevation, as is seen in other oak-feeding, elevation-restricted Lepidoptera in the region (C. Schmidt pers. comm.). Habitats at these localities vary somewhat and include Madrean oak woodland and oak grasslands. We are unaware of collections of this moth in higher elevation oak-pine woodland habitats. *Cicinnus chambersi* is a typical summer monsoon moth, flying from early July to early August. Mimallonid larvae take several months to mature in North America, therefore those interested in locating the larvae should look for mature larvae on oaks in the autumn.

#### Distribution.

**Figure 24.**
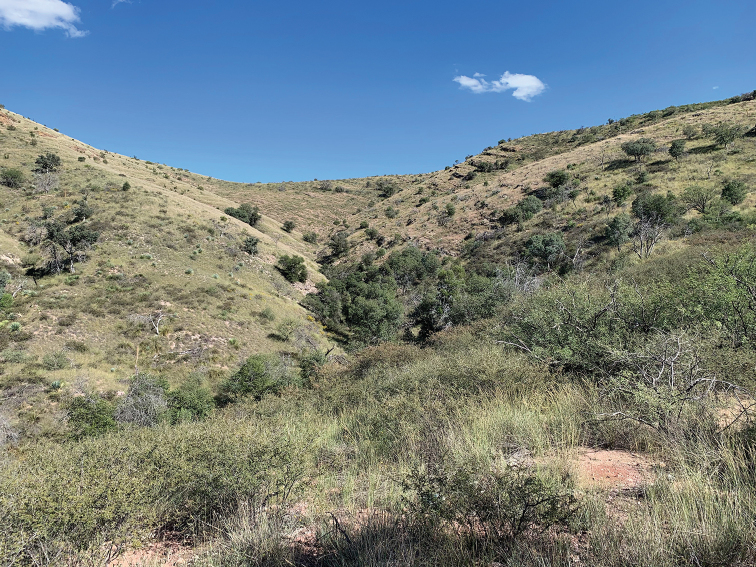
Type locality of *Cicinnus chambersi*, Arizona, Santa Cruz Co., Peña Blanca Lake, Pajarito Mtns., Coronado National Forest. Photo courtesy of Aaron Chambers.

**Figure 25.**
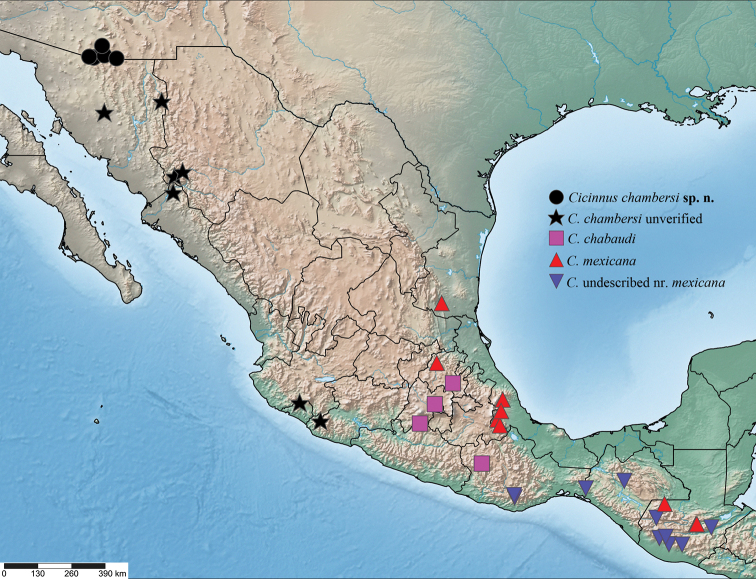
Map depicting localities of *C. chambersi* and species related to *C. mexicana* examined in the present study. See the Suppl. material 3: File S1 for full data for all points.

*Cicinnus chambersi* is known only from sky island mountain ranges of southeastern Arizona (Figs 24, 25). The type series is restricted to Arizona material, but undoubtedly *C. chambersi* occurs in mountain ranges with similar oak-dominated habitats in northwestern Mexico. See below for a more in-depth discussion of additional specimens from Mexico. *Cicinnus chambersi* has been collected in the Huachuca, Pajarito, Patagonia, and Santa Rita Mountains, with the northernmost observation being Box Canyon on the northern edge of the Santa Rita Mountains (Pima County).

#### Etymology.

This new species is named for Aaron Chambers of Tucson, Arizona, a desert dweller and dear friend of the authors, in recognition of his support of native biodiversity and for imparting his expansive ecological knowledge of the Sonoran Desert to us every monsoon season.

#### Remarks.

It is surprising that *C. chambersi* has been overlooked in North America due to the distinct morphological differences between *C. chambersi* from Arizona and the common *C. melsheimeri* with which the new species has been confused. However, *C. chambersi* appears to be a rarely collected moth considering the few specimens known to us and the intensity at which insect collecting occurs in southern Arizona, and this could be the reason *C. chambersi* has not yet been described. To our knowledge, no specimens of *C. chambersi* were available to Franclemont at the time of his 1973 revision (St Laurent pers. obs. of the Cornell University Insect Collection). In fact, we are not aware of any specimens collected in the United States prior to 2009. Therefore, it is also possible that *C. chambersi* is a relatively recent establishment from Mexico in southern Arizona.

Apart from the differences between *C. chambersi* and *C. melsheimeri* revealed by genitalia dissections, ongoing phylogenomic work using anchored hybrid enrichment (Lemmon et al. 2012), in which hundreds of conserved loci have been sequenced for nearly all described genera of Mimallonidae, have shed light on the relative relationships of species within *Cicinnus*. *Cicinnus melsheimeri*, *C. chambersi* and its putative sister species, C. cf. mexicana, have been included in the phylogenomic work of St Laurent et al. (2020). In these authors’ study (see their fig. 2), *C. melsheimeri* is sister to the remainder of *Cicinnus*, with C. cf. orthane (type species of *Cicinnus*) sister to *C. chambersi* + C. cf. mexicana (all relationships with 100% support). This topology is supported by our morphological study, in that *C. melsheimeri* is the most distinct *Cicinnus* species and is the only one to bear vincular arms (Fig. 17), with all other known *Cicinnus* species lacking them. Our barcoding efforts carried out for the present study have revealed what are essentially identical topological relationships (Fig. 1) as in St Laurent et al. (2020), albeit with lower UFBoot support than in the phylogenomic work which utilizes much more substantial, genomic, datasets. Regardless, important takeaways are illustrated by the COIML tree here, which recovers *C. melsheimeri* sister to all other *Cicinnus*; with the cicinnine *Gonogramma hanseni* (Herbin & Monzón, 2015) used to root this tree. The holotype and a paratype of *C. chambersi* have been barcoded and are presented in the tree in Fig. 1 in this work; and form a clade sister to two other Central American *Cicinnus*: *C. melgibsoni* Herbin & Monzón, 2015 and C. cf. solvens Dyar, 1914. This clade together is sister to a densely sampled *C. mexicanasensu lato* clade which also includes the Central American *C. tuisana* Schaus, 1910, a species remarkably similar in external appearance to *C. mexicana*. We recognize that two distinct entities exist under the name *C. mexicana* (which together are not monophyletic), with the clade containing topotypical *C. mexicana* from Veracruz, Mexico being here considered *C. mexicanasensu stricto.* True *C. mexicana*, therefore, is sister to *C. tuisana*, a similar species found in Costa Rica and Panama, in our COIML analysis. The other clade is unnamed at this time pending ongoing taxonomic work of the genus; but it suffices to say that morphology, phylogenomic, and mitochondrial evidence all support a closer relationship between the newly described *C. chambersi* and Central American *Cicinnus* species, than to the unique, largely North American *C. melsheimeri*.

##### *Cicinnus* in Mexico, further discussion

In describing *C. chambersi*, it is necessary to go into some additional depth in discussing the *Cicinnus* of Mexico in order to couch the new species within the broader context of its conspecifics in the region, where several described and undescribed taxa are found. The common North American *C. melsheimeri*, discussed above in the diagnosis of *C. chambersi* and in the phylogenetic justification for the validity of the new species, also appears to be found in Mexico. *Cicinnus melsheimeri* is found throughout the eastern United States and southeastern Canada, with sparse records in the Rocky Mountains and the western United States (Colorado, Utah, northern Arizona, central and northern New Mexico, and the Big Bend Region of western Texas, and southern Texas) (St Laurent unpublished). Additional populations of a taxon near *C. melsheimeri* are known from throughout the mountainous regions of Mexico (in Chihuahua, Distrito Federal, Hidalgo and Nuevo Leon) as well, these specimens display the typical genitalia of *C. melsheimeri* complete with the vincular arms (NHMUK dissection NHMUK010402293 of a specimen from Hidalgo, examined). The degree of cryptic diversity included under the name *C. melsheimeri* is yet to be fully resolved, though this will be a worthy area of research. It is clear however, that true *C. melsheimeri* (type locality, Pennsylvania, USA), which we here consider to include all populations morphologically most similar to the eastern USA species, as well as those in Mexico, can be readily differentiated from the remainder of *Cicinnus* (including *C. chambersi*) by the vincular arms that are present in the male genitalia. *Cicinnus melsheimeri* has been shown to represent a distinct lineage of *Cicinnus* sister to the remainder of the genus within a phylogenomic (St Laurent et al. 2020) and mitochondrial barcoding context (this paper).

Other *Cicinnus* populations in Mexico, including *C. chambersi* which ranges as far north as southern Arizona, belong to the more typical, primarily tropical American group of *Cicinnus* that always lack vincular arms in the male genitalia. This putative species-group contains all *Cicinnussensu stricto* (*sensu*St Laurent and Kawahara 2019) except *C. melsheimeri*. In Mexico, only three named species are known: *C. chabaudi* (Figs 9, 18), *C. melgibsoni*, and *C. mexicana* (Figs 14, 20). *Cicinnus chabaudi* is restricted to arid south-central Mexico in the vicinity of Distrito Federal (extending at least to northwestern Oaxaca as per a specimen in CRAS). *Cicinnus melgibsoni* was described from Guatemala but is also found in southern Mexico (Herbin and Monzón 2015). The widespread *C. mexicana* was described from Orizaba, Veracruz, Mexico. The first author has examined numerous *C. mexicana* from near the type locality of this species as well as throughout Mexico, Guatemala, and Belize. Preliminary morphological studies and barcoding conducted herein suggest that there are at least two putative species under the name *C. mexicana*, but they do not form a monophyletic group. One putative species, *C. mexicanasensu stricto*, is found in eastern and southern Mexico, on the eastern slopes of the Sierra Madre Oriental into central Guatemala (Baja Verapaz); and a second, undescribed, species occurs in southern Mexico (Chiapas and Oaxaca) and southern Guatemala. We do not describe the southern populations as a new taxon here, pending ongoing studies of *Cicinnus*, as it will be necessary to include additional populations. For example, the Costa Rican species *C. tuisana* also falls into this broader *C. mexicanasensu lato* clade (Fig. 1) introducing additional uncertainty about the identity of these taxa. Despite these issues however, *C. chambersi* is morphologically, genetically, and biogeographically distinct from any of these other taxa.

In northwestern Mexico there exists no name to adequately refer to *Cicinnus* species there, except for specimens clearly more allied to *C. melsheimeri* as discussed previously. Therefore, *C. chambersi* is the first named species belonging to the typical Neotropical *Cicinnus* species-group described from the arid southwestern United States and (likely) northwestern Mexico. We are aware of five specimens of *Cicinnus* from northwestern Mexico that are morphologically similar to *C. chambersi* but are from scattered localities with inadequate numbers of specimens from each location to allow for a convincing determination as *C. chambersi*. These specimens were also not barcoded. Therefore, none of these specimens are included in the type series of *C. chambersi* in order to conservatively restrict the type series to specimens collected at and around the type locality in southern Arizona. Each of the five northwestern Mexican specimens will be discussed below in order to bring attention to them in hopes that additional material will be discovered or collected to better determine their identities. Complete collecting data, including institutional depositions, for these specimens can be found in the supplemental appendix (Suppl. material 3: File S1).

One male specimen from the AMNH bears a label reading “Horcasitas.” We believe this refers to San Miguel de Horcasitas in Sonora, though admittedly the data are poorly documented. Externally this specimen resembles *C. chambersi* but has straighter forewing margins and is in otherwise poor condition. The genitalia differ from *C. chambersi* with the valvae of the Horcasitas specimen more rounded and curved upwards, which is more typical of *C. mexicana*.

In the BME there are two apparently conspecific male specimens from Chihuahua, one from Temoris and another from Cuiteco, and a putatively conspecific female from Choix, Sinaloa. While these Chihuahuan males are externally very similar to *C. chambersi* from Arizona, and inhabit comparable habitats, the female from nearby Choix displays a postmedial line angled differently from any examined female *C. chambersi*, and thus makes the determination of the Chihuahuan males as *C. chambersi* inconclusive (if the two males and the female are regarded as conspecific).

A single large male from the AMNH with the following data is also worth discussing: Mexico, Sonora, Mile 6.2, Colonia Mesa Tres Ríos to Huachinera. This specimen is larger and paler than any examined *C. chambersi*, and from farther east than any other putative Mexican *C. chambersi*.

Finally, a single male from Minatitlán, Colima (in VOB) and a single female from nearby Michoacán (Barcode of Life Datasystems, BC-Her2624) are known, which are morphologically more similar to *C. chambersi* than *C. mexicana* and may represent another undescribed species near *C. chambersi* or the southern extent of the distribution of this species. We were unable to examine the genitalia of this population, though a barcode of the Michoacán specimen places it sister to *C. chambersi* (see Fig. 1) in our analysis, supporting the hypothesis that they are closely related or perhaps even conspecific with *C. chambersi*. We hope that additional collecting in these regions will help elucidate the distribution of *C. chambersi* in Mexico, as well as to clarify the specific identities of the abovementioned specimens from Chihuahua, Colima, Michoacán, Sonora, and Sinaloa.

## Supplementary Material

XML Treatment for Cicinnus chambersi

## Supplementary materials

Supplementary material 1 Figure S1 Data type PDF figure Explanation note Maximum likelihood phylogenetic tree inferred with IQ-TREE based on the COI marker, rooted to *Gonogramma hanseni*. https://binary.pensoft.net/file/405479This dataset is made available under the Open Database License (http://opendatacommons.org/licenses/odbl/1.0/). The Open Database License (ODbL) is a license agreement intended to allow users to freely share, modify, and use this Dataset while maintaining this same freedom for others, provided that the original source and author(s) are credited.Ryan A. St Laurent, Lawrence E. Reeves, Akito Y. Kawahara

Supplementary material 2 Table S1 Data type Species data Explanation note Data for Cicinnini specimens used in this study, either sequenced *de novo* or extracted from publicly available data on BOLD (http://www.boldsystems.org/). https://binary.pensoft.net/file/405480This dataset is made available under the Open Database License (http://opendatacommons.org/licenses/odbl/1.0/). The Open Database License (ODbL) is a license agreement intended to allow users to freely share, modify, and use this Dataset while maintaining this same freedom for others, provided that the original source and author(s) are credited.Ryan A. St Laurent, Lawrence E. Reeves, Akito Y. Kawahara

Supplementary material 3 File S1 Data type Species data Explanation note Data and remarks for additional *Cicinnus* examined in this study. https://binary.pensoft.net/file/405481This dataset is made available under the Open Database License (http://opendatacommons.org/licenses/odbl/1.0/). The Open Database License (ODbL) is a license agreement intended to allow users to freely share, modify, and use this Dataset while maintaining this same freedom for others, provided that the original source and author(s) are credited.Ryan A. St Laurent, Lawrence E. Reeves, Akito Y. Kawahara

Supplementary material 4 File S2 Data type Genetic data Explanation note FASTA file of COI alignment for all taxa used in Figure 1/ Supplementary material 1. https://binary.pensoft.net/file/405482This dataset is made available under the Open Database License (http://opendatacommons.org/licenses/odbl/1.0/). The Open Database License (ODbL) is a license agreement intended to allow users to freely share, modify, and use this Dataset while maintaining this same freedom for others, provided that the original source and author(s) are credited.Ryan A. St Laurent, Lawrence E. Reeves, Akito Y. Kawahara

Supplementary material 5 File S3 Data type Phylogenetic data Explanation note Tree file in NEWICK format corresponding to phylogeny in Figure 1/ Supplementary material 1. https://binary.pensoft.net/file/405483This dataset is made available under the Open Database License (http://opendatacommons.org/licenses/odbl/1.0/). The Open Database License (ODbL) is a license agreement intended to allow users to freely share, modify, and use this Dataset while maintaining this same freedom for others, provided that the original source and author(s) are credited.Ryan A. St Laurent, Lawrence E. Reeves, Akito Y. Kawahara
