# Supplementary figures and images for: Cicinnus chambersi: a new species of sack-bearer moth (Lepidoptera, Mimallonidae, Cicinninae) from southeastern Arizona, USA
Source: Zookeys. 2020 Apr 30;931:49–71. doi: 10.3897/zookeys.931.50203 (PMC7205858; doi:10.3897/zookeys.931.50203)

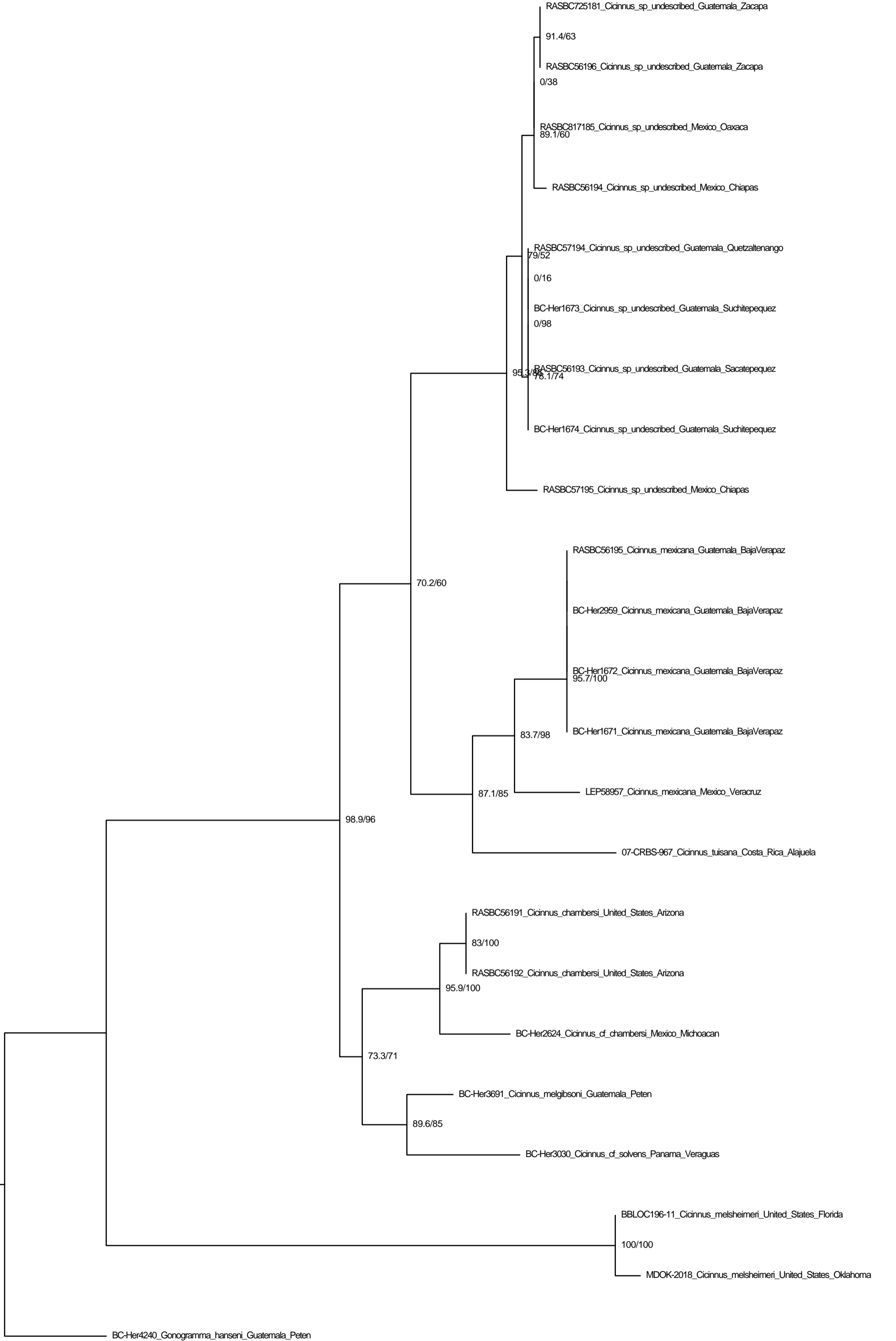

0.03

Supplement: Supplementary material 1 — Figure S1 [file zookeys-931-049-s001.pdf]
