# Supplementary material for: Cicinnus chambersi: a new species of sack-bearer moth (Lepidoptera, Mimallonidae, Cicinninae) from southeastern Arizona, USA
Source: Zookeys. 2020 Apr 30;931:49–71. doi: 10.3897/zookeys.931.50203 (PMC7205858; doi:10.3897/zookeys.931.50203)
Supplement: Supplementary material 3 — File S1 [file zookeys-931-049-s003.docx]

**Supplementary material 3**

**Data and remarks for additional *Cicinnus* examined in this study.**

***Cicinnus melsheimeri* (Harris, 1841)**

**Type material. Syntype. UNITED STATES OF AMERICA** – **Pennsylvania** • ♂; M.C.Z. Type 26381/ 776 ♂ Penn./ *Perophora melsheimeri* Harr. Type/ (MCZ).

**Material examined [Arizona, New Mexico, and Mexico populations only].** (5 ♂, 5 ♀ total) **MEXICO** – **Chihuahua** • 1 ♂; Cuiteco; 22.VII.1969; T.A. Sears, R.C. Gardner, C.S. Glaser [leg.]; (BME). – **Hidalgo** • 4 ♂, 1 ♀; Guerrero Mill, Hidalgo; 9000 ft [2,743 m]; Mann & Skewes [leg.]; Rothschild Bequest BM 1939-1, NHMUK 010896103, 010896104, 010896107, NHMUK dissections 010402293 [male], 010402294 [female] (2 ♂, 1 ♀, NHMUK), USNM-Mimal: 1558, 1559 (2 ♂, USNM). – **UNITED STATES OF AMERICA** – **Arizona** – **Coconino County** • 1 ♀; Williams; “VI-20-36”; Grace H. and John I. Sperry [leg.]; (AMNH). – **Yavapai County** • 1 ♀; Prescott; 26.VII.1926; O.L. Poling [leg.]; (CUIC). – **New Mexico** – **Otero County** • 1 ♀; High Rolls; 32°56'52.69''N, 105°50'21.06''W; 6500' [1,981 m]; 27.V.2012; E. Rand leg; (Coll. E. Rand, Arizona). – **Texas** – **Cameron County** • 1 ♀; Brownsville; late May 1989; J. Adams leg; (MGCL

**Remarks.** *Cicinnus melsheimeri* is a widespread species in the eastern United States but occurs sporadically in the Rocky Mountains southwards into northern and central Arizona and New Mexico. There are specimens from northwestern and central Mexico as well, and preliminary genitalia examination suggests that Mexican populations of *C. melsheimeri* may represent a distinct species, albeit very closely related to *C. melsheimeri*. Given the expansive distribution of this species, however, we do not perform any additional species descriptions at this time. Other specimens in the AMNH that we did not examine firsthand come from Distrito Federal and Hidalgo, Mexico.

We list these specimens here as they were mentioned in the main text and also because it is noteworthy that *C. chambersi* and *C. melsheimeri* may in fact be sympatric in parts of Mexico (namely in Chihuahua).

***Cicinnus chambersi* – putatively identified, Mexican specimens**

**Material examined.** (5 ♂, 1 ♀ total) **MEXICO** – **Chihuahua** • 1 ♂; Temoris; 19.VII.1969; T.A. Sears, R.C. Gardner, C.S. Glaser [leg.]; (BME) • 1 ♂; Cuiteco; 29.VII.1969; T.A. Sears, R.C. Gardner, C.S. Glaser [leg.]; (BME). – **Colima** • 1 ♂; Minatitlán; 19°26'N, 103°57'W; 2100 m; 14.VI.2000; V.O. Becker Col.; Col. Becker 121879 (VOB). – **Michoacán** • 1 ♀; Environs de Coalcoman, Puerto La Zarzamora, Cerro; 18.7977°, -103.227°; 1635 m; 27.VI.2008; Haxaire & Paquit [leg.]; BOLD sample ID: BC-Her2624 (collection of D. Herbin, on BOLD). – **Sinaloa** • 1 ♀; Choix; 6.VII.1968; T.A. Sears, R.C. Gardner, C.S. Glaser [leg.]; (BME). – **Sonora** • 1 ♂; Horcasitas [San Miguel de Horcasitas]; “15.III.”; C.C. Hoffman [leg.]; St Laurent dissection: 8-10-18:2 (AMNH) • 1 ♂; Mile 6.2, Colonia Mesa Tres Ríos to Huachinera; 5900 ft [1,798 m]; VII.1.1979; R. Holland [leg.]; St Laurent barcode: 5-7-19:1 [unsuccessful] (AMNH).

**Remarks.** Each of the above specimens is addressed directly in the main text as these are considered as being possible Mexican examples of *C. chambersi*; however, only the female from Michoacán has been barcoded. Although this barcoded specimen forms a clade with the two barcoded types of *C. chambersi* from Arizona, it is reasonably distinct (in terms of branch length and biogeographically) to cause some doubt for conspecificity, although it is clearly the most closely related barcoded specimen of all those examined from outside of Arizona.

***Cicinnus chabaudi* Dyar, 1914**

**Type material. Holotype.** **MEXICO** – **Distrito Federal** • ♂; Zacualpan, Mexico/ RMuller Collector/ June 13/ 3775/ USNM-Mimal: 1136/ Type No. 16503 U.S.N.M./ *Cicinnus chabaudi* Type Dyar/ (USNM, examined).

**Additional material examined.** (3 ♂ total) **MEXICO** – **Distrito Federal** • 1 ♂; Los Lomas; VII.1950; T. Escalante; A.C. Allyn Acc. 1973-48, UF FLMNH MGCL 1031579 (MGCL). – **Hidalgo** • 1 ♂; Guerrero Mill; 9000 ft [2,743 m]; (NHMUK). – **Oaxaca** • 1 ♂; ca. 15 km SE San Martín Huamalulpan, Cabañas Yucunuvichi; 17°19.71'N, 97°38.09'W; 2200 m; 22.V.2015; C. Conlan, S. Naumann, & B. Wenczel leg.; St Laurent dissection: 8-10-18:3 (MGCL).

**Remarks.** *Cicinnus chabaudi* is a rarely collected species endemic to drier south-central Mexico but is included in the present article as it shares an apparent close relationship with other Mexican *Cicinnus* species namely based on male genitalia (St Laurent dissection: 8-10-18:3, Fig. 18) as this species has not yet been sequenced for AHE or barcoding.

We are also aware of four additional specimens of *C. chabaudi* in the AMNH, but we have only incomplete data for them: two males from Mexico, one male from Distrito Federal, and a male from Michoacán.

***Cicinnus mexicana* (Druce, 1898), *sensu stricto***

**Type material. Syntype. MEXICO** – **Veracruz:** • ♀; Orizaba (USNM, examined).

**Additional material examined.** (43 ♂, 9 ♀ total) **GUATEMALA** – **Baja Verapaz** • 2 ♂; Purulhá; 1620 m; 20.VII.2000; V.O. Becker col.; Col. Becker 123090; (VOB) • 1 ♂; Purulhá; Ranchitos del Quetzal lodge; [15.216017°, -90.2191°]; 1600 m; “6/10/08”; (CRAS) • 3 ♂; SE Purulhá, Ranchitos de Quetzal, Parque Ecológico Gucumatz (cloud forest); N15°12.961' W90°13.146' [15.216017°, -90.2191°]; 1660 m; 1.X.2016; J. Monzón, S. Naumann & H. Schnitzler leg.; St Laurent dissection: 7-25-18:4; (1 ♂ CRAS, 2 ♂ MGCL ) • 13 ♂, 2 ♀; Quetzal Res., Los Ranchitos; 1680–1700 m; 10–15.VI.2007; J.B. Heppner [leg.]; (2 ♂, MGCL); 19–22.IX.2008; J.B. Heppner [leg.]; UF FLMNH MGCL 1031772, 1031781, 1031785 [♀], 1031810 (5 ♂, 1 ♀, MGCL); 11–16.X.2011; J.B. Heppner [leg.]; UF FLMNH MGCL 1031768; (6 ♂, MGCL); • 3 ♂; Quetzal Res., “Los Rancheros”; 1650 m:; 10–12.X.2005; J.B. Heppner [leg.]; (2 ♂, MGCL); 21–23.IX.2006; J.B. Heppner [leg.]; UF FLMNH MGCL 1031771 [♀]; 1031776; (1 ♂, 1 ♀ MGCL) • 2 ♂, 1 ♀; Quetzal Res., Los Ranchitos, 1680 m; 12–14.VI.2013; J.B. Heppner [leg.]; UF FLMNH MGCL 1031800; St Laurent barcode: 5-6-19:5; (1 ♂, 1 ♀, MGCL); 3–6.VI.2017; J.B. Heppner & E. Fuller [leg.]; (MGCL) • 1 ♂; 6 km E. Purulhá; [15.230725°, -90.178288°]; 3–4.VII.1992; P. Hubbell [leg.]; UF FLMNH MGCL 1031757 (MGCL) • 1 ♀; Km 156 on Rd. to Cobán; 14.V.1991; P. Hubbell [leg.]; UF FLMNH MGCL 1031769 (MGCL). –**Huehuetenango** • 1 ♂; Barillas, Unión Las Palmas; 15.9311°, -91.29931°; 1,444 m; 28.V.2011; Camposeco & Monzón Col.; (CJM). – **MEXICO** – **Chiapas** • 1 ♂; East of La Trinitaria; 16.114721°, -91.729721°; 30.X.2019; photograph on iNaturalist.org, not collected. – **Hidalgo** • 1 ♂; 30 mi. SW Chapulhuacan; 24.VI.1966; J.S. Buckett M.R & R.C. Gardner coll; (BME). – **Nuevo Leon** • 1 ♂; Monterrey; 25.550058°, -100.270202°; 28.IX.2015; (photograph on iNaturalist.org, not collected). – **Tamaulipas** • 1 ♂; Gómez Farias; 2200 m; 29.V.1997; V.O. Becker col.; Col. Becker 109245; (VOB) • 1 ♀; Gómez Farias; 1000 m; 31.VII.1988; V.O. Becker, M. A. Solis [leg.]; Col. Becker 69210; (VOB) • 1 ♂; Gómez Farias; (CUIC). – **Veracruz** • 3 ♂, 1 ♀; Huatusco; 1500 m; 19–23.VIII.1981; V.O. Becker col.; Col. Becker 43837 (VOB) • 2 ♂, 1 ♀; Huatusco; 1300 m; 19–23.VIII.1981; V.O. Becker col.; Col. Becker 43837, USNM-Mimal: 2085; (USNM) • 1 ♂; Instituto Tecnologico Superior de Zongolica; “behind campus and by the river”; 18.6502°, -97.0079°; 27.VIII.2017; Kawahara & Toussaint coll.; DNA voucher # LEP-58957; (MGCL) • 1 ♀; Huatuxco; (NHMUK) • 4 ♂; Jalapa [Xalapa]; (3 NHMUK; 1 USNM) • 3 ♂; Orizaba; (2 NHMUK; 1 USNM).

**Remarks.** The above specimens were identified as *C. mexicana* based on proximity to the type locality of this species (eastern Mexico, in the states of Hidalgo, Nuevo Leon, Tamaulipas, and Veracruz) and external morphology; as well as barcoding results for specimens from Baja Verapaz, Guatemala. These specimens are also nearly identical to those found in eastern Mexico. The specimen in CJM from Huehuetenango, Guatemala has not been barcoded, but is from a locality near to the Baja Verapaz population, and matches *C. mexicana* morphologically, namely in the straight forewing margins and deeper, redder coloration, especially submarginally on the forewings.

***Cicinnus* sp*.* undescribed, near *C. mexicana***

**Material examined.** (37 ♂, 5 ♀ total) **MEXICO** – **Chiapas** • 1 ♂; Hwy 190, 5 km E. of Rizo del Oro; 16°28'04.91''N, 94°01'44.44''W [16.468031°, -94.029011°]; 820 m; 14.V.2015; S. Naumann & B. Wenczel leg.; St Laurent barcode: BC 5-7-19:5; (CRAS) • 2 ♀; San Cristobal de las Casas env., nr. Hotel Flores; 16°43'42.10''N, 92°41'43.32''W [16.728361°, -92.695367°]; 2,415 m; 15.V.2015; S. Naumann & B. Wenczel leg.; St Laurent barcode: 5-6-19:4; (1 ♀, CRAS); 20.V.2015; C. Conlan, S. Naumann, & B. Wenczel leg.; (1 ♀, CRAS). – **Oaxaca** • 3 ♂; rd. Oaxaca to Puerto Angel, S. San José del Pacifico, Restaurante Lupita; 16°09'19.96''N, 96°29'44.86''W [16.155555°, -96.496234°]; 2460 m; 13.V.2015; S. Naumann & B. Wenczel leg.; St Laurent dissection and barcode: 8-17-18:5; (CRAS) • 1 ♂; Portillo de Rayo; 16°14'N 96°31'W [16.233333°, -96.516667°]; 1750 m; V.2013; ex. coll. S. Naumann (CRAS). – **GUATEMALA** –– **Huehuetenango** • 1 ♂; Bulej; 15°27'N, 91°35'W; 2,000 m; 25.VII.2000; V.O. Becker leg.; Col. Becker 123533; (VOB). – **Quetzaltenango** • 1 ♂; Fuentes Georginas; N14°45.012', W91°28.823' [14.750099°, -91.480328°]; 2,418 m; 26.IX.2016; J. Monzón, S. Naumann, & H. Schnitzler leg.; St Laurent barcode: 5-7-19:4 (CRAS). – **Sacatepéquez** • 1 ♂; SE Antigua, San Cristobal El Bajo, Finca El Pilar, Cerro Cucurucho; N14°31.192', W90°41.467' [14.519867°, -90.691135°]; 2,620 m; 22.IV.2017; J. Monzón & S. Naumann leg.; St Laurent barcode: 5-6-19:3 (CRAS). – **Suchitepéquez** • 1 ♂; Volcan Atitlán, Los Terrales coffee plantation (seasonal rainforest); N14°32.040', W91°08.853' [14.534009°, -91.147544°]; 1045 m; 27.IX.2016; leg. J. Monzón, S. Naumann & H. Schnitzler leg.; St Laurent dissection and barcode: 7-25-18:3; (CRAS). – **Sololá** • 1 ♂; Bosq. Corazon [Corazón Del Bosque], Novilliero; [14.790256°, -91.261078°]; 2,435 m; 13–15.V.2017; J.B. Heppner & E. Fuller [leg.]; (MGCL) • 1 ♂; Corazon Bosque [Corazón Del Bosque], Novilliero; [14.790256°, -91.261078°]; 2,400 m; 12–13.VI.2015; J.B. Heppner & E. Fuller [leg.]; St Laurent barcode: 5-6-19:7 [unsuccessful]; (MGCL) • 4 ♂; Bosq. Corazon [Corazón Del Bosque], Novilliero; 2,435 m; 13–15.V.2017; J.B. Heppner & E. Fuller [leg.]; (MGCL) • 2 ♂; Los Tarrales, Volcán Atitlán; 800–1000 m; 24–26.IX.2006; J.B. Heppner [leg.]; UF FLMNH MGCL 1031805 (MGCL). – **Zacapa** • 8 ♂; Sierra de las Minas, N Rio Hondo, E San Lorenzo, Cerro Monos env; N15°06.971' W89°40.764' [15.116183, -89.679400]; 2,243 m; 1–2.V.2017; J. Monzón & S. Naumann leg.; St Laurent dissection and barcode number 7-25-18:1 (see Figs 13, 19); (6 ♂, CRAS; 2 ♂ MGCL with DNA voucher # LEP-51269 – note: among these specimens are morphotypes in line with both *C. mexicana sensu stricto* and the undescribed species); additional St Laurent dissections in CRAS from this locale: 8-17-18:3, 8-17-18:4 • 1 ♂, 1 ♀; Sierra de las Minas, San Lorenzo; 1,730 m; 29–30.IX.2008; J. Heppner, B. Sutton, & G. Steck [leg.]; St Laurent barcode: 5-6-19:6; “pines”; (MGCL) • 2 ♂, 1 ♀; Sierra de las Minas, San Lorenzo; 1,645 m; 1–3.X.2008; J. Heppner, B. Sutton, & G. Steck [leg.]; UF FLMNH MGCL 1031796 [♀]; “oaks”; (MGCL) • 1 ♂; Sierra de las Minas, San Lorenzo, 1645 m; 1–3.X.2008; J. Heppner, B. Sutton, & G. Steck [leg.]; “oaks”; (MGCL). – **BELIZE** – **Cayo** • 7 ♂, 1 ♀; Mtn. Pine Ridge; 1000’ Falls; 25.V.1990 [2 ♂], 30.V.1990 [1 ♀], 26.VI.1990 [3 ♂], 29.VI.1990 [2 ♂]; Linwood C. Dow (MGCL) • 1 ♂; Augustine, Mt. Pine Ridge; 500 m; 24–25.IX.1973; V.O. Becker col., Col. Becker 21389 (VOB).

**Remarks.** Based on barcoding results (see main text Fig. 1), specimens from southern Mexico (states of Chiapas and Oaxaca) and southern Guatemala are conspecific and are not reciprocally monophyletic with respect to *C. mexicana sensu stricto* from eastern Mexico and Baja Verapaz, Guatemala. Not all populations listed above for this undescribed taxon could be verified with barcoding (namely those from Sololá and Huehuetenango, Guatemala and from Belize). In general, this undescribed species is lighter in coloration, with less red suffusion on the dorsum of the forewings, though some specimens (particularly certain specimens from Zacapá, Guatemala) are indistinguishable from *C. mexicana sensu stricto* from Baja Verapaz, Guatemala; and therefore, may be more appropriately identified as *C. mexicana sensu stricto*, and their determination here as an undescribed species is preliminary and based on barcoding results of some specimens from Zacapá. However, the two morphotypes from Zacapá have not both been barcoded, only the lighter specimens have been barcoded. Both morphotypes were collected at the same locality and on the same dates. One specimen (a photograph on iNaturalist.org) from southeastern Chiapas is considered above to be more in line with *C. mexicana sensu stricto* based on external morphology, but clearly specimens from this region are of rather uncertain determination.

Genitalia among Mexican *Cicinnus* species is homogenous, with intraspecific variation in otherwise useful characters (gnathos and juxta structures, for example), and therefore coloration and distribution are currently more useful general metrics for differentiating *C. mexicana* and the undescribed taxon from southern Mexico and Guatemala, pending a full revision of *Cicinnus*.
